# Compartment Syndrome of the Hand Presenting as Bullous Edema

**DOI:** 10.7759/cureus.7290

**Published:** 2020-03-16

**Authors:** Phillip R Braunlich, Kate Braunlich, Ryan Brink, Risa Ross, John N Harker

**Affiliations:** 1 Orthopedic Surgery, Largo Medical Center, Largo, USA; 2 Dermatology, Largo Medical Center, Largo, USA; 3 Internal Medicine, Largo Medical Center, Largo, USA

**Keywords:** bullae, compartment syndrome, hand, edema, fasciotomy

## Abstract

Compartment syndrome of the hand can be a challenging diagnosis to ascertain. The difficulty in diagnosis is in part due to the absence of an established diagnostic criteria. Additionally, when a patient presents obtunded or with an altered sensorium, the identification of compartment syndrome of the hand can be further complicated. Despite the potential difficulty in diagnosis, it is of upmost importance for orthopedic surgeons to recognize and treat this entity in an expeditious manner. Without prompt treatment, the risk is increased morbidity including possible amputation. Here, we present a unique and thought-provoking case along with a review of the literature. The purpose of sharing this case is to highlight potential clues to aid in prompt diagnosis and improve patient outcomes.

## Introduction

Compartment syndrome occurs when interstitial pressure within a compartment prevents the tissue within from receiving adequate perfusion [1-3]. Ashton observed that when a critical threshold pressure is met, vessel collapse occurs as interstitial pressure exceeds that of intraluminal pressure. Venous congestion follows, ultimately leading to reduced perfusion and tissue necrosis [4]. Despite much literature on compartment syndrome of the lower extremity and forearm, the same cannot be said of that of compartment syndrome of the hand. Perhaps part of the reason is compartment syndrome of the hand is a perplexing diagnosis and one that does not have a set of concrete diagnostic criteria [3]. Expedient recognition is necessary for the best possible outcome, as early identification may prevent muscle necrosis, contractures, and amputation [5-7]. Some of the most common causes include trauma (i.e. fractures and crush injuries), snake envenomation, high-pressure injections, intravenous drug use, insect bites, and intravenous (IV) fluid extravasation [1,8]. Currently, the signs used to diagnosis compartment syndrome of the hand include extent of swelling, palpation of tense compartments and intensity of pain [5]. Another clue is the hand typically assumes an intrinsic minus position (metacarpophalangeal (MCP) joint extension, proximal interphalangeal (PIP) and distal interphalangeal (DIP) flexion; claw hand) [9]. This diagnosis becomes even more difficult in a patient with an altered sensorium, such as those on mechanical ventilation. This scenario removes the ability of the patient to provide a history, mechanism of injury, or quantify pain on examination. At this point, the next step is measurement of compartment pressures, which will provide objective data [10]. While the compartments of the extremities have a universal threshold value of within 30 mmHg from the diastolic blood pressure or a measured absolute value of equal to or greater than 30 mmHg for the diagnosis of compartment syndrome, the same cannot be said about the hand. A review of the current literature reveals much debate regarding a strict diagnostic value for compartment syndrome of the hand. Regardless, it is imperative for the orthopedic surgeon to maintain a high level of clinical suspicion of compartment syndrome [8]. Delayed or missed compartment syndrome has a devastating impact on the functional outcome of the patient, leading to necrosis, contractures and possibly the loss of the extremity [5-7].

## Case presentation

**Figure 1:**
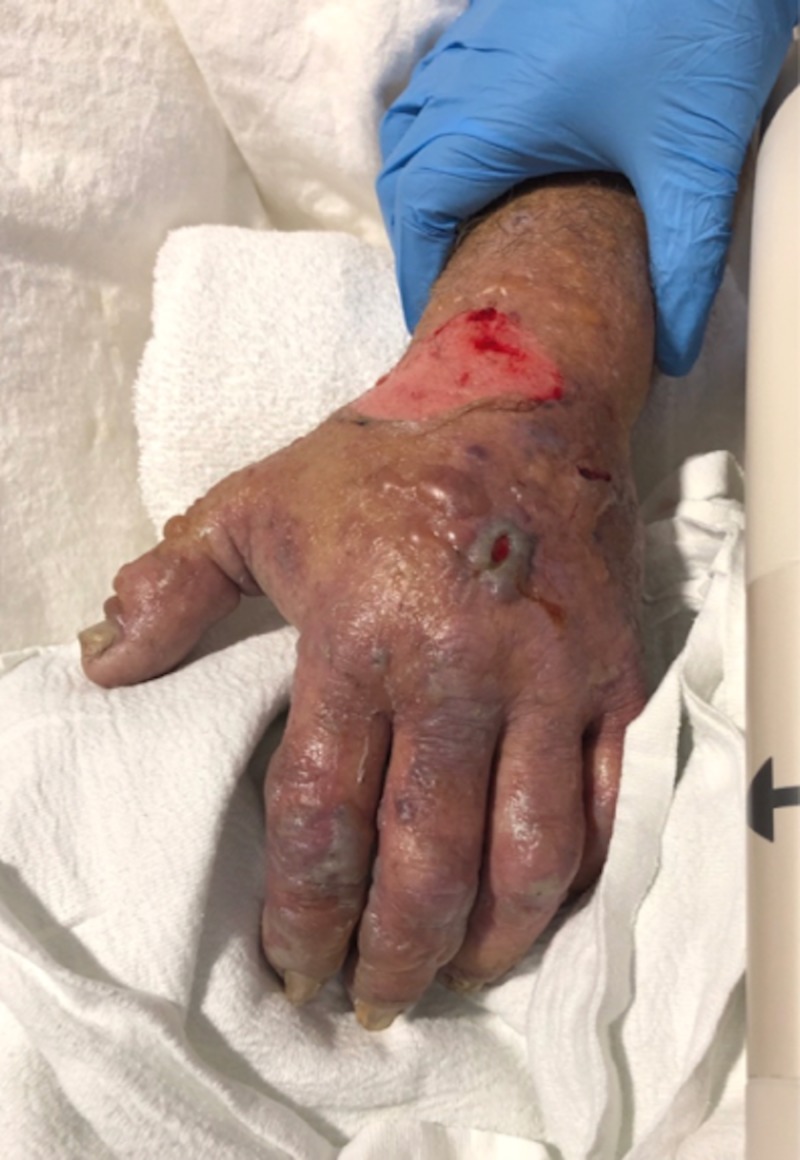
Edematous, erythematous to violaceous left hand

**Figure 2:**
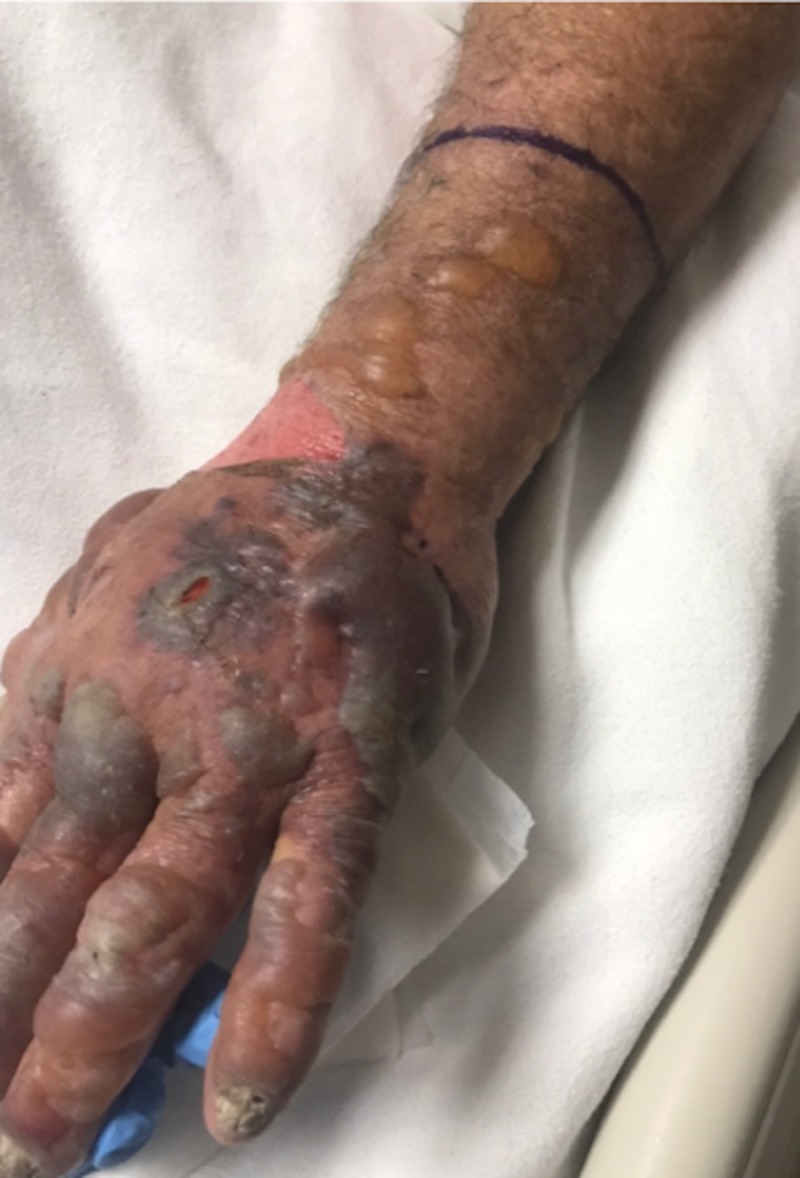
Edematous, erythematous to violaceous left hand and forearm

**Figure 3:**
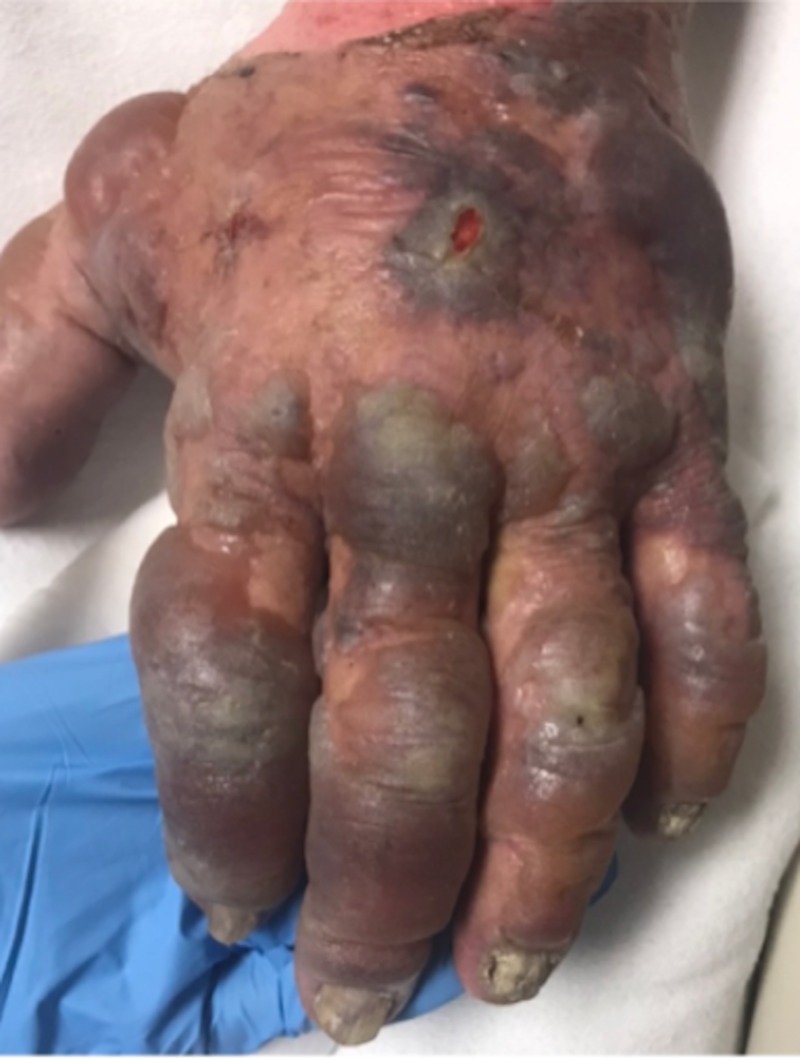
Hemorrhagic and serous bullae on the dorsal hand with a 2-cm superficial ulcer

**Figure 4:**
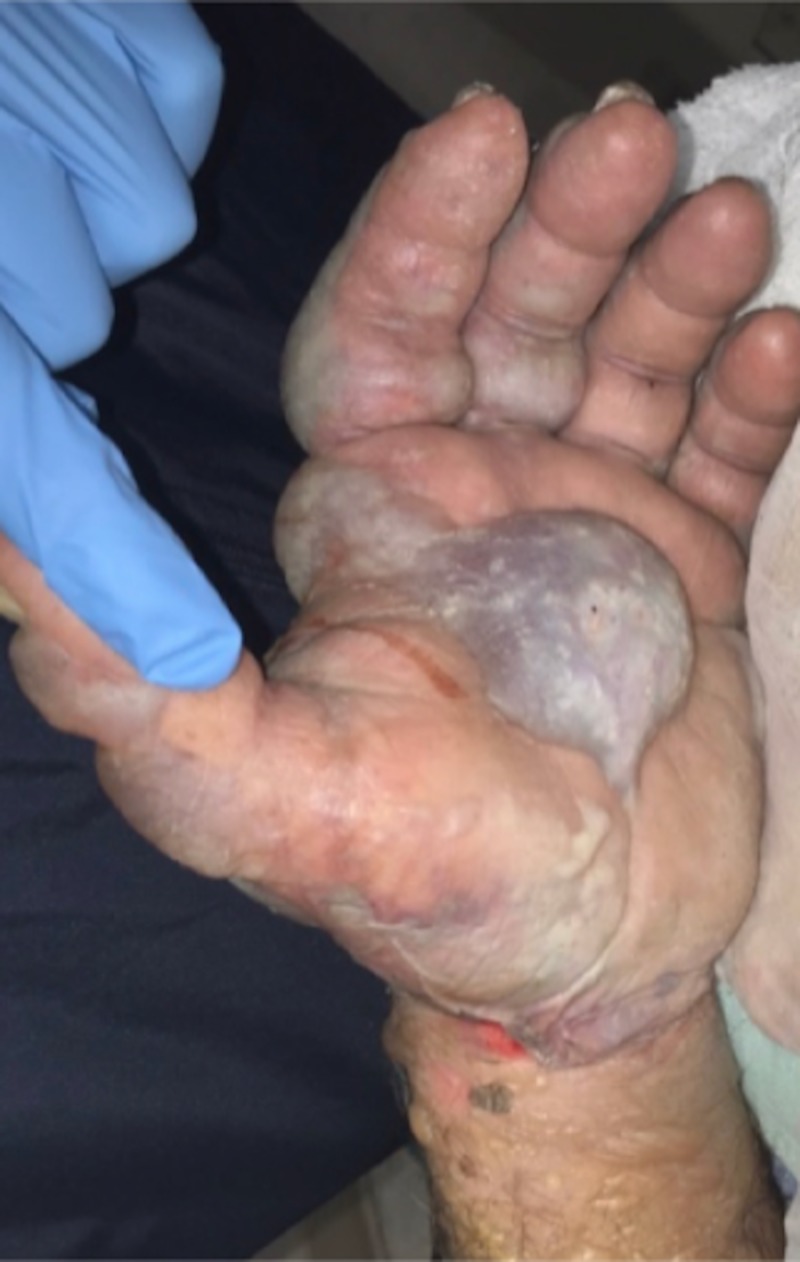
Hemorrhagic and serous bullae on the palmar hand

A 60-year-old male was admitted for routine sigmoidectomy secondary to multiple unresectable colonic polyps. Surgery was complicated by significant blood loss, conversion to open hemicolectomy, and eventual transfer to the intensive care unit on mechanical ventilation. Dermatology and orthopedic surgery were consulted on post-operative day 2 and 3, respectively, for bullous edema of the left hand. Gross findings are demonstrated in Figures 1-4. Dermatology preformed punch biopsies of the bullous lesions and orthopedic surgery performed compartment pressure measurements using a Stryker needle device. The thenar and hypothenar compartments were found to have a pressure of 75 mmHg and the remaining compartments of the hand had pressures <20 mmHg. At this time, the decision to proceed with 10-compartment fasciotomy was made. Interestingly, the fourth dorsal interossei compartment musculature appeared non-viable and the hypothenar and thenar compartments had intra-compartmental hematoma, but viable musculature.

## Discussion

The importance of prompt diagnosis and treatment of compartment syndrome is paramount for positive patient outcomes. However, there is currently an absence of universally accepted diagnostic signs and compartment pressures for the diagnosis of compartment syndrome of the hand. Missed compartment syndrome or delay of emergent fasciotomy has devastating impact on the functional outcome of the patient, leading to necrosis, contractures and even amputation [5-7]. Table 1 summarizes the disparity of absolute compartment pressures in the literature. Whitesides et al. proposed that an absolute pressure above 50 mmHg was necessary for muscle necrosis to begin. However, other authors have hypothesized differing absolute pressures and even proposed delta compartment pressures (difference of the measured compartment pressure from the diastolic blood pressure) as the most accurate objective diagnostic tool [8,10-12]. Due to ambiguity in the current literature, it is crucial to take other objective and subjective findings into account. We suggest acute bullous eruption as a presenting symptom of compartment syndrome of the hand. Bullous edema is indicative of severe injury to the underlying soft tissue and may herald the development of increasing compartment pressures. This is particularly important for patients unable to quantify pain or mechanism of injury. The presence of tense compartments on physical exam, in conjunction with bullous edema, should prompt the clinician to measure compartment pressures or consider emergent fasciotomy. We believe the presence of bullous edema aids in the diagnosis of acute compartment syndrome of the hand and should be considered in the diagnostic criterion. Nevertheless, more research is needed to establish a consensus regarding absolute compartment pressures to make this important diagnosis.

**Table 1:** Intra-compartmental pressures indicative of underlying compartment syndrome of the hand Shown here is the wide variation evident in our current literature.

TABLE 1	
Whitesides et al. [10]	50 mmHg
Matava et al. [11]	20 mmHg within diastolic BP
Naidu et al. [12]	30 mmHg
Ouellette et al. [7]	15-25 mmHg

## Conclusions

It is imperative to have a high level of clinical suspicion for compartment syndrome of the hand in patients with unexplained edema and hemorrhagic bullae. While an innovative set of diagnostic criteria and a consensus threshold of compartment pressures remains to be published, the available clinical history and clinical gestalt must allow the orthopedic surgeon to make a quick decision regarding treatment. Without prompt intervention, poor outcomes should be expected and are not limited to just necrosis and contractures, but possibly loss of limb or life.
